# Aquaculture 4.0: hybrid neural network multivariate water quality parameters forecasting model

**DOI:** 10.1038/s41598-023-41602-7

**Published:** 2023-09-26

**Authors:** Elias Eze, Sam Kirby, John Attridge, Tahmina Ajmal

**Affiliations:** 1 School of Architecture, Computing and Engineering, University of East London, University Way, London, E16 2RD UK; 2 Institute for Research in Applicable Computing (IRAC), School of Computer Science and Technology, University of Bedfordshire, Vicarage St, Luton, LU1 3JU UK; 3 Chelsea Technologies Ltd, 55 Central Avenue, West Molesey, Surrey, KT8 2QZ UK

**Keywords:** Ocean sciences, Mathematics and computing

## Abstract

This study examined the efficiency of hybrid deep neural network and multivariate water quality forecasting model in aquaculture ecosystem. Accurate forecasting of critical water quality parameters can allow for timely identification of possible problem areas and enable decision-makers to take pre-emptive remedial actions that can significantly improve water quality management in aquaculture industry. A novel hybrid deep learning neural network multivariate water quality parameters forecasting model is developed with the aid of ensemble empirical mode decomposition (EEMD) method, deep learning long-short term memory (LSTM) neural network (NN), and multivariate linear regression (MLR) method. The presented water quality forecasting model (shortened as EEMD–MLR–LSTM NN model) is developed using multivariate time-series water quality sensor data collected from Loch Duart company, a Salmon offshore aquaculture farm based around Scourie, northwest Scotland. The performance of the novel hybrid water quality forecasting model is validated by comparing the forecast result with measured water quality parameters data and the real Phytoplankton data count from the aquaculture farm. The forecast accuracy of the results suggests that the novel hybrid water quality forecasting model can be used as a valuable support tool for water quality management in aquaculture industries.

## Introduction

Harmful Algal Blooms (HAB) is a global issue, spanning across oceans, rivers, lakes, and ponds, especially with regarding aquaculture industry. Many countries worldwide have documented occurrences of HABs, and their frequency may increase due to global warming and human impact on marine environments. The international community is alarmed by HABs because they not only endanger human health and marine ecosystems but also have a detrimental impact on local and regional economies. Studies have shown that precision aquaculture system can be adopted for early detection of HAB and allow ample time for aquafarmers and decision-makers to take precautionary measures^1,2^.

A precision aquaculture system requires an efficient system for quick decision making using continuous water quality parameter data^1,2^. However, continuous and accurate water quality parameters measurement using conventional methods is usually an expensive and labour-intensive process^3^. With the conventional sampling and testing techniques, aquaculture water quality parameters are usually approximated through laboratory analyses which are both expensive and time-consuming. These conventional aquaculture water quality monitoring and management techniques involve the collection of water samples from the relevant site of interest, water sample storage and transportation to the laboratory, as well as chemical tests and analysis at the laboratory. All these processes from water sample collection to laboratory analysis require the usage of expensive equipment and a fair amount of time before obtaining the actual water quality parameter results. In the course of these tedious and time-consuming processes, room for inefficiency and error usually arises^4^. This delays any corrective measures that can be taken. A precision aquaculture system relies not only on sophisticated online water quality monitoring of farm but using the data to design early warning systems^5^. If the water quality parameters dataset is automatically monitored and analysed through the artificial neural network (ANN) technique.

Research has shown that the adverse effects of aquaculture water quality pollution can be efficiently tackled with the automation of water quality parameters dataset analysed and timely prediction of water quality^2^. Therefore, it is essential to devise new aquaculture water quality data variation trends analysis and forecasting approaches and methodologies to promote high productive aquaculture businesses. Several studies have attempted to devise ways of coping with water quality contamination using both conventional numerical modelling methods, least squares support vector regression (LSSVR), NNs methods like Radial Basis Function NN (RBFNN), Back Propagation NN (BPNN) algorithms, and machine learning methods to forecast future water quality changes^6–9^. However, addressing the seasonal variation of aquaculture water quality for high yield aquaculture industry, a temporal dimension to the data analysis must be considered to guarantee an effective and efficient aquaculture water quality parameters dataset analysis and prediction of future water quality parameters. Hence, multivariate statistical approaches such as Principal Component Analysis (PCA) has been applied to determine relationship among various water quality parameters^10^. These geo-statistical approaches that have been applied include multivariate interpolation, multiple linear regression analysis, transitional probability, kriging, etc.^11^. Some of the algorithms applied for water quality parameters dataset analysis and forecasting also include Artificial Intelligence (AI) approaches such as Bayesian Networks (BN)^12^, Support Vector Regression (SVR)^13^, Neuro-Fuzzy Inference^10^, Decision Support System (DSS), Auto-Regressive Moving Average (ARMA)^14^, hybrid Sparse Auto-encoder (SAE) and LSTM (SAE-LSTM), SAE and BPNN (SAE-BPNN)^15^, and piecewise multivariate imputation (PWIMP) method^16^. However, the challenge with traditional numerical and geo-statistical approaches, LSSVR, NNs such as RBFNN and BPNN techniques is the inherent weakness of long-term dependency problem. Similarly, research has shown that the non-linear nature of water quality parameters dataset makes it rather complicated to map input/output (I/O) dataset and forecast future water quality parameters^17^. But further studies have shown that deep learning long-short term memory (LSTM) NN can overcome the above-mentioned weakness and can provide efficient applicability and reliability for aquaculture water quality parameter prediction^18–22^. Additionally, combining ensemble empirical mode decomposition (EEMD) method with deep learning LSTM NN has demonstrated clear advantages over traditional LSTM NNs in terms of improved water quality parameter prediction accuracy in the aquaculture environment^9,21^.

In seeking solution to the above-mentioned challenges associated with tackling the prevailing water quality contamination in aquaculture industry, more research must be done in areas of effectiveness, efficiency, prediction accuracy, reliability and usability of the existing water quality prediction models and management methodologies in the precision aquaculture ecosystem. In this study, a novel hybrid deep learning-based forecasting model for aquaculture industry is proposed. The proposed forecasting model combines the EEMD and multivariate regression methods to decompose, learn the temporal dimensional features of the measured water quality parameters dataset signals, and establish a relationship among the different parameters before applying deep learning LSTM NN to predict the water quality. This will allow the decision-makers in aquaculture industry to better understand and manage water contamination in aquaculture environment and improve the farm productivity. In this paper we present the design of a precision aquaculture system that monitors water quality data at an aquaculture site (Loch Duart) using a dedicated Multi-parameter Trilux sensor developed by Chelsea Technologies Ltd which monitors and measures only three key Algal parameters such as Chlorophyll-a (measured at two different excitations—CHL470 and CHL530), and Turbidity. This data forms the basis for the multivariate prediction model that can predict the occurrence of HAP events at the Salmon aquafarm.

The rest of the paper is organised as follows. Section presents the "Methods and materials". Section discussed the "Multivariate linear regression" method applied in this study. Section "Proposed hybrid forecasting model design" contains the proposed novel hybrid EEMD–MLR–LSTM NN model design. Section presents the "Performance evaluation metrics". Section contains the "Results and discussions", while Section “Conclusion” concludes the paper.

## Methods and materials

### Study area description, aquaculture dataset acquisition and analysis

**Figure 1:**
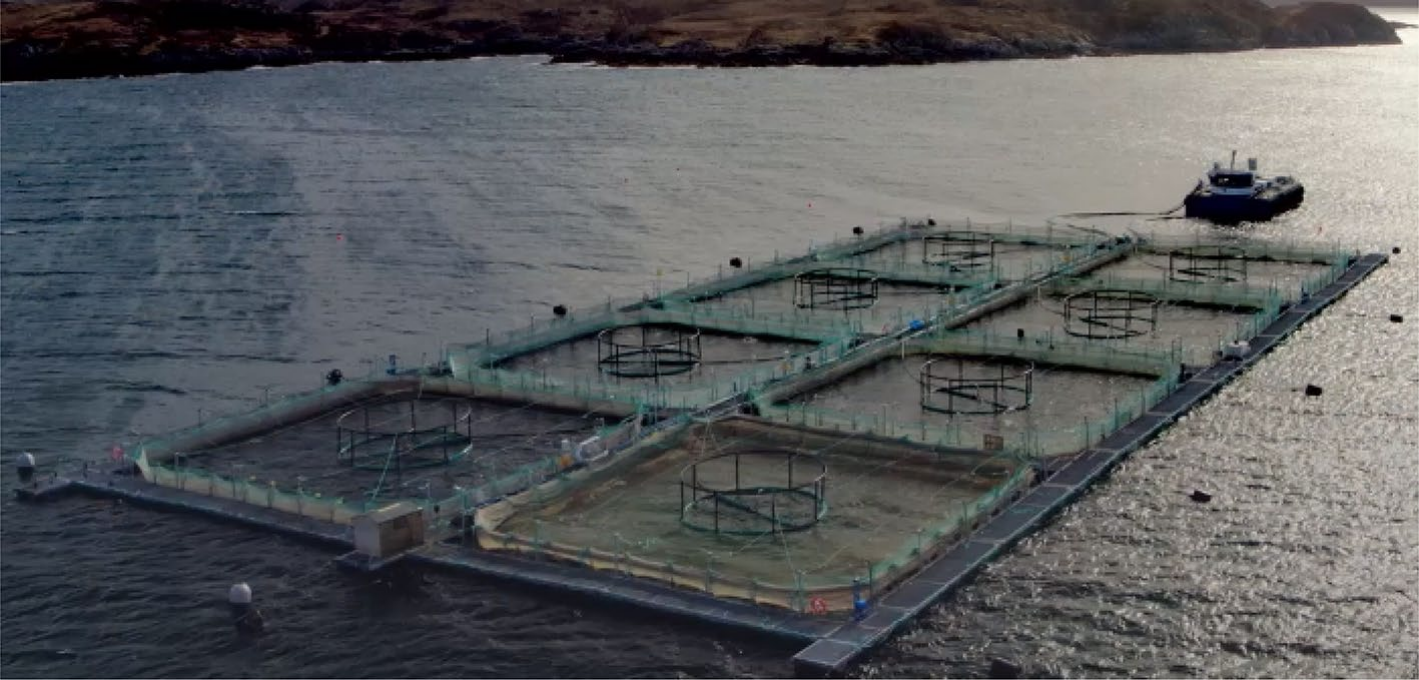
The eight (8) sea-sites at the Loch Duart salmon aquaculture farm.

**Figure 2:**
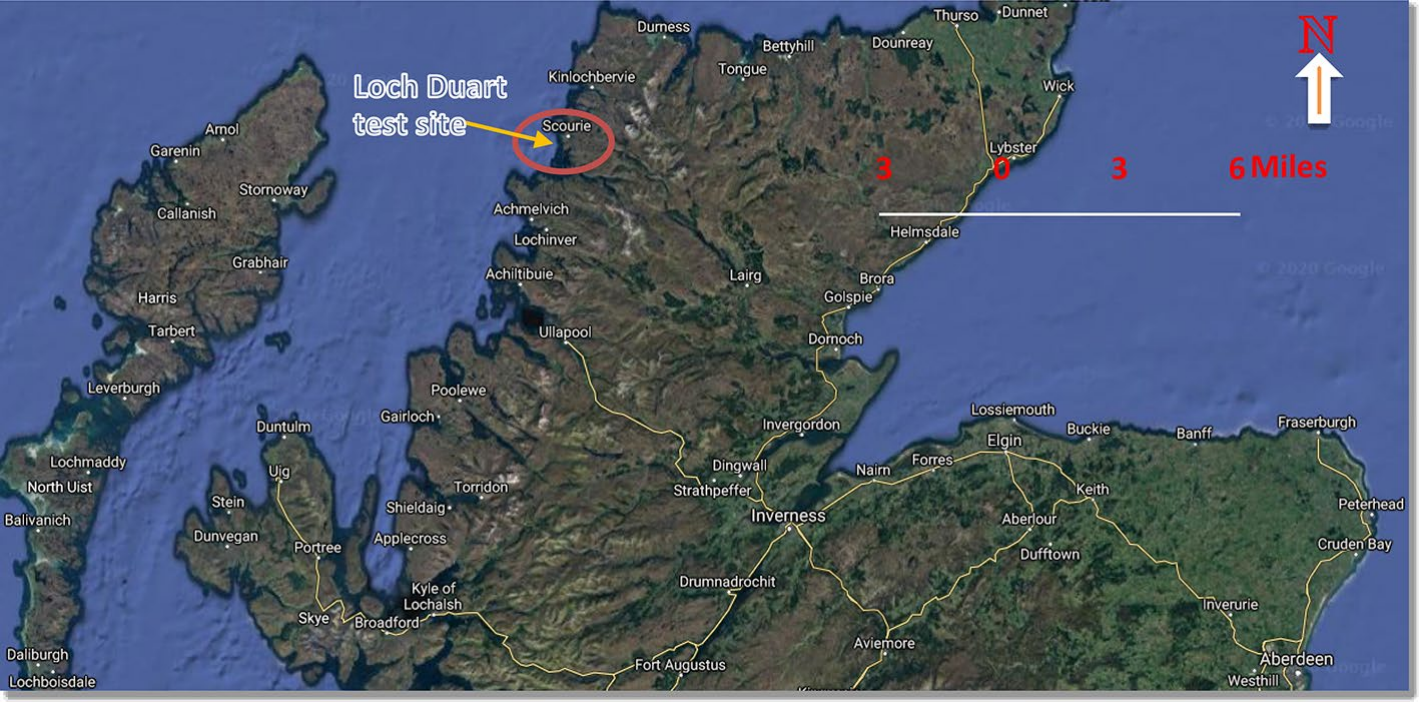
Chelsea Technologies’ multi-parameters Trilux sensor deployment site—Loch Duart Salmon offshore aquaculture farms based around Scourie, northwest Scotland^24^.

Loch Duart is an independent Scottish salmon aquafarm industry, which has its’ headquarter in Scourie, Sutherland in north-west Scotland. The Salmon farming company owns and operates 8 sea-sites (see Fig. 1) and 2 hatcheries in Sutherland and the Outer Hebrides. In Loch Duart, Salmon are hatched and grown in the cold, clear freshwater of North-west Scotland. The salmon farming company annually harvests approximately 5000 tons of fresh salmon. Chlorophyll-a (µg/L) measured at two different excitations (CHL470 and CHL530) and Turbidity time series data were collected with the aid of a TriLux multi-parameter sensor probe, a 3-in-1 fluorometer designed and developed by Chelsea Technologies Ltd^23^. The sensor deployment took place at one of their sheltered sites along the coast. The sensor probes installation location is depicted in Fig. 2 and equipped with solar powered telemetry system to allow for remote data transmission to cloud platform for storage and analysis. The telemetry unit was secured to the metal walkway around the outside of the net pens and the sensor was situated on the outside of one of the outermost pens, nearest to the feed barge. Table 1 shows the list of other sensors developed by Chelsea Technologies Ltd and the corresponding parameters that each of them monitors.

**Table 1:** Chelsea Technologies Ltd Fluorometers/sensors and parameters monitored^26^.

	Fluorometers							Active fluorometers					Optical sensors		
	UniLux	TriLux	UviLux	VLux AlgaePro	VLux TPro	VLux FuelPro	VLux OilPro	LabSTAF	FastOcean APD	FastOcean	Act2 Lab	FastBallast	PAR Sensor	GlowTracka	UniLux Turbidity
*Fluorometers*															
Chlorophyll-a	×	×		×	×	×	×	×	×	×	×	×			
Phycobiliproteins	×	×		×	×	×	×	×	×	×	×	×			
Fluorescein	×														
Rhodamine	×														
BTEX			×			×									
PAH			×				×								
Tryptophan			×		×										
CDOM			×		×	×	×								
*Active fluorometers*															
Variable Fluorescence								×	×	×	×	×			
Fluorescence Light Curves (FLC)								×		×					
Phytoplankton Primary Productivity								×	×	×					
Phytoplankton Cell Counting												×			
*Optical sensors*															
PAR													×		
Bioluminescence														×	
Turbidity	×	×		×	×	×	×								×
Absorbance					×	×	×								

**Figure 3:**
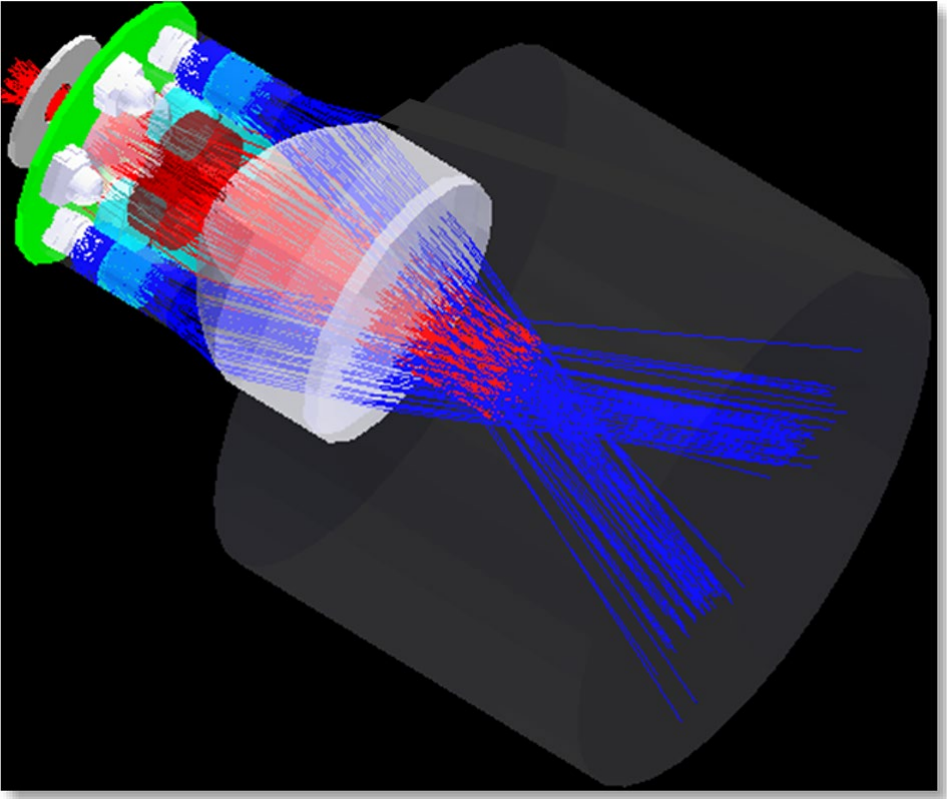
Chelsea Technologies’ TriLux multi-wavelength fluorometer with solar powered telemetry system.

**Figure 4:**
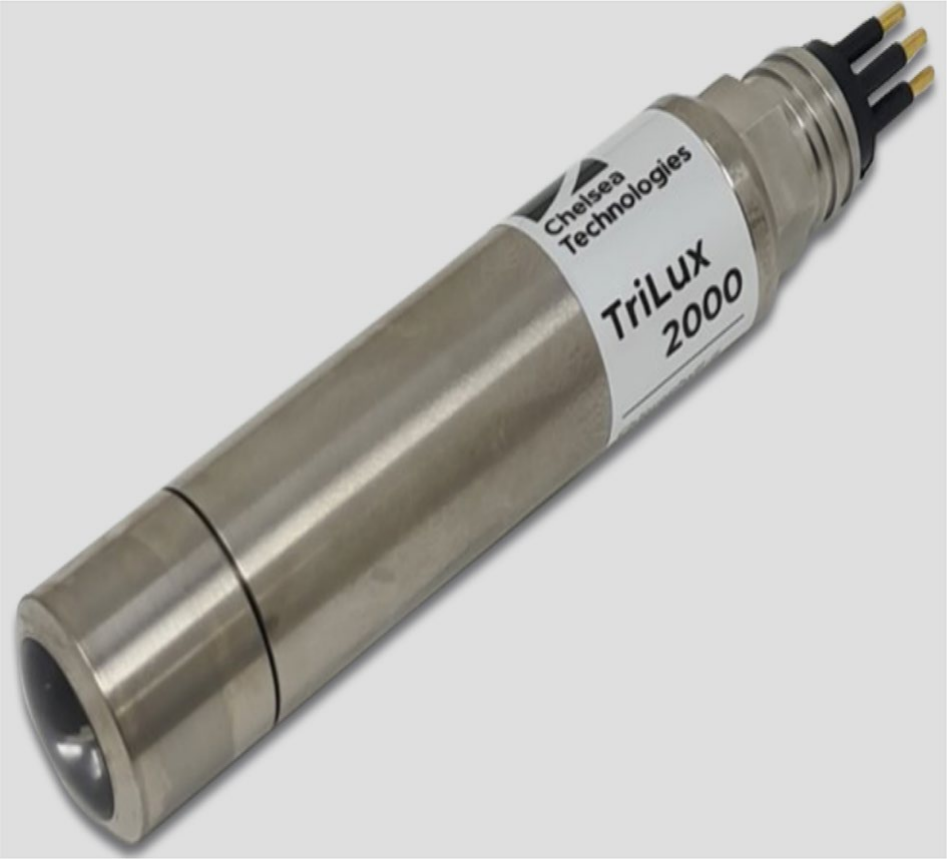
Chelsea Technologies’ TriLux multi-parameter fluorometer which monitors three key algal parameters in a single probe^24^.

The TriLux multi-parameter fluorometer (see Figs. 3 and 4) was used for monitoring and collection of a total of 22,708 sets of non-stationary, non-linear water quality parameters time-series data at Loch Duart Salmon aquafarm between May and October 2020. This TriLux multi-parameter fluorometer is a low cost, compact sensor that monitors three key algal parameters in a single, highly sensitive probe. The 3-in-1 fluorometer allows for widespread water quality monitoring in a variety of applications, including harmful algal blooms, aquaculture, water treatment works, river catchments and coastal studies^25^. These water quality parameters are Chlorophyll-a (470 nm), Turbidity, and Chlorophyll-a (530 nm).

**Figure 5:**
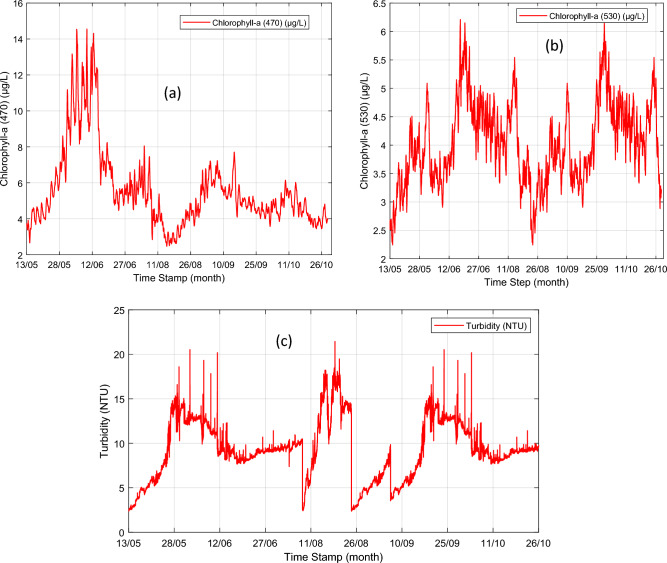
The trend variation of the time-series contents of: (**a**) CHL470 (µg/L), (**b**) CHL530 (µg/L), and (**c**) Turbidity.

At the Loch Duart offshore aquaculture farms, water quality dataset for the three parameters is collected every ten (10) minutes using the TriLux sensor. The collected time series dataset for Chlorophyll-a (470), Chlorophyll-a (530), and Turbidity parameters using Chelsea Technologies’ TriLux multi-parameter sensor are plotted as line graphs in Fig. 5a–c which show the water quality trends variations between May and October 2020. Although, Fig. 5a and b show that there are some erratic trend variations of Chlorophyll-a (470) (mg/L) and Chlorophyll-a (470) (mg/L), respectively, between May and October 2020, Fig. 5c show that most outliers were seen in the time-series data trend variations of Turbidity between May and October 2020. Further investigation by the sensor installation team from Chelsea Technologies Ltd revealed that the high presence of outliers as seen in the sensor-measured time-series data was caused by biofouling incident which adversely affected the TriLux sensor readings. The biofouling challenge was immediately resolved through the integration of a low-cost wiper to the installed Chelsea Technologies’ multi-parameter algal fluorometer at the study cite of Loch Duart Salmon aquafarm in Scotland. For our study, the already collected time-series datasets were cleaned through pre-processing, while filling of missing data was done as described in 2.3, and outliers removed by a function of the hybrid model that detects and removes outliers.

Generally, the 470 channel measures chlorophyll fluorescence from direct excitation of chlorophyll-a that usually strongly correlate with phytoplankton biomass in freshwater. On the other hand, CHL530 channel measures Chlorophyll fluorescence from the excitation of an accessory pigment that is found in cyanobacteria. Under normal conditions with no cyanobacteria present, there is likely to be a low level of CHL530 fluorescence emission that tracks with the CHL470 emission because Chlorophyll-a does absorb some green light (at 530 nm). Therefore, when there is a cyanobacteria bloom occurrence in the aquaculture water body, it is expected to notice a divergence in these signals. The high correlations between these three data signals and the cyanobacteria in freshwater, as shown in Section "Data correlation analysis" is used in devising a multivariate linear regression equation that can be applied to determine the presence of harmful green biomass (Algae) bloom occurrence in the Scottish salmon aquaculture water body.

### Data pre-treatment, filling and correction

Non-linear, non-stationary water quality parameters time-series dataset defects usually result in excessive deviation between the measured original water quality parameters values and the forecast results. The basis of accurate time-series analysis and the development of effective and reliable predictive models is high-quality sample data. To provide concise, accurate dataset for the forecasting model and improve forecasting accuracy, the measured water quality parameters dataset was carefully pre-processed. Usually, the issue of missing data is inevitable with automatic water quality sensor monitoring systems. In this case, the water quality parameters such as Chlorophyll-a (470), Chlorophyll-a (530), and Turbidity were automatically measured throughout the days and nights at 10 min’ intervals. To fill in any missing data, filling-in approach called linear interpolation algorithm^27^ is applied to achieve a better estimation effect that can accurately approximate the missing data values. In data analysis, linear interpolation algorithm takes the ratio of two known data-points and one unknown data-point as a linear relationship. Therefore, to obtain the missing, unknown water quality parameter value, linear interpolation technique applies the slope of the presumed line to compute the time-series dataset increment. Hence, the dataset is completed.

#### Definition 1.

The nature of the measured parameters.

An installed automated freshwater TriLux multi-parameters sensor monitoring system at the Loch Duart Salmon offshore aquaculture farms measures time series water quality parameters at a constant time interval everyday which can be denoted as $\beta$, then $n$ length time-series of the measured water quality parameters datasets is defined as (1);

1 $${S}_{i,n}=\left\{\left({X}_{i, 1},{T}_{1}\right),\left({X}_{i, 2},{T}_{2}\right), \ldots ,\left({X}_{i, n},{T}_{n}\right)\right\}$$

where ${X}_{i, l}$ represents the value of the measured ${i}^{th}$ time-series water quality factor by the automatic sensory monitoring system at time ${T}_{l}$ $\left(1\le i\le \beta , 1\le l\le n\right)$, and at other given ${T}_{l}$, the time interval is constant at $\Delta T=\left({T}_{l+1}-{T}_{l}\right)=10$ minutes. Therefore, if the original value ${X}_{i, l}$ is missing, its estimated value ${\widehat{X}}_{i, l}$ can be obtained with the problem of minimum which is given as $\left|{\widehat{X}}_{i, l}-{X}_{i, l}\right|$ changed into the missing value estimation problem. Based on the measured data ${X}_{i, x}$ and ${X}_{i, y}$ at time ${T}_{i, x}$ and ${T}_{i, y}$, respectively, the linear imputation function $L\left(t\right)$ could be formulated for the time series water quality parameters sensor monitoring system as:

2 $$L\left(t\right)={X}_{i, x}+\left(\frac{{X}_{i, x}-{X}_{i, y}}{{T}_{i, x}-{T}_{i, y}}\right)\cdot \left(t-{T}_{i, x}\right).$$

For any missing time series water quality parameters data at any given moment, the linear interpolation algorithm firstly finds the two closest moments ${T}_{i, x}$ and ${T}_{i, y}$ $\left({T}_{i, x}<t<{T}_{i, y}\right)$, and estimates the lost data value at time $\mathrm{t}$ with the help of the known measured data ${X}_{i, x}$ and ${X}_{i, y}$ of ${T}_{i, x}$ and ${T}_{i, y}$ moments based on Eq. (2), i.e., ${\widehat{X}}_{n}=L\left(t\right)$.

### Data correlation analysis

**Figure 6:**
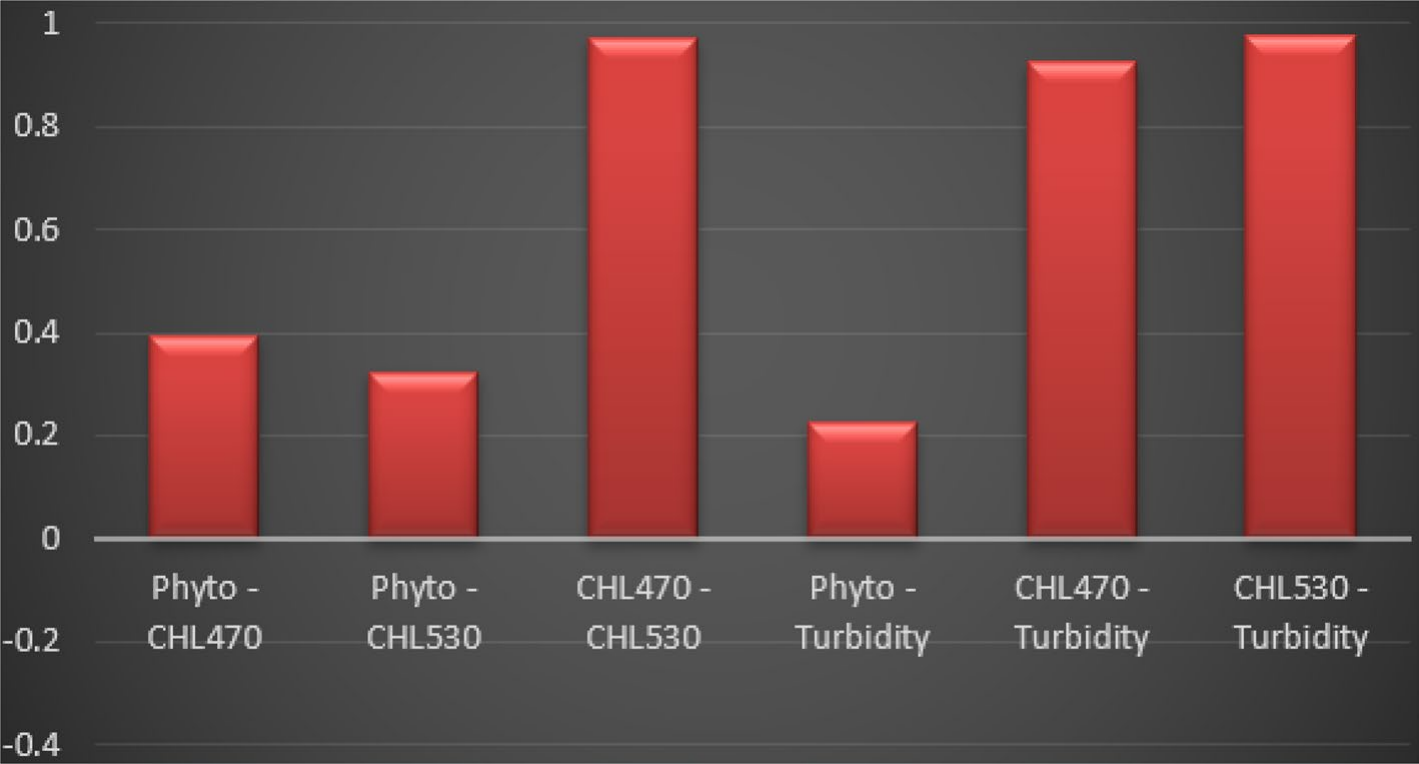
Correlations graphs plot of the measured Chlorophyll-a (470), Chlorophyll-a (530), Turbidity, and the Phyto data count.

This study applied the Pearson’s correlation coefficient technique to analyse the existing correlations between the TriLux multi-parameters sensor measured time series aquaculture water quality parameters such as Chlorophyll-a (470), Chlorophyll-a (530), Turbidity, and the Phytoplankton data count at the Loch Duart Salmon offshore aquaculture farms. To better understand the existing correlations between two variables, the Pearson’s correlation coefficient technique^28^ has been widely used as a data analysing technique, which is also described as the quotient of co-variance and standard deviation between two variables. The Pearson’s correlation coefficient system was used after cleaning and pre-processing the TriLux multi-parameters sensor measured time series water quality parameters, to analyse the existing correlations between the required parameters. Table 2 contains the correlations between the measured Chlorophyll-a (470), Chlorophyll-a (530), Turbidity, and the Phytoplankton data count obtained through data analysis and calculations for the months of May and June of 2020. Similarly, Fig. 6 shows the plotted correlations graphs of the measured Chlorophyll-a (470), Chlorophyll-a (530), Turbidity, and the Phytoplankton data count.

**Table 2:** Data correlation analysis result.

	CHL70	Turbidity	CHL530	Phytoplankton
CHL70	1			
Turbidity	0.925066	1		
CHL530	0.972895	0.974055	1	
Phytoplankton	0.394222	0.223068	0.32407	1

Both Table 2 and Fig. 6 show that the three monitored and measured water quality parameters have a positive correlation with the green biomass (Phytoplankton) presence in the freshwater. These clearly indicate that while CHL470, CHL530, and Turbidity show a positive correlation with Phytoplankton, CHL470 maintains an extremely positive correlation with CHL530. This is expected because the 470 channel measures Chlorophyll fluorescence from direct excitation of Chlorophyll-a that usually strongly correlate with the presence of Phytoplankton biomass in freshwater. Similarly, 530 channel measures Chlorophyll fluorescence from the excitation of an accessory pigment that is generally present in Cyanobacteria—which is a specie of Phytoplankton. In other words, under normal conditions, where there is absence of Cyanobacteria in the freshwater, there is likely to be an extremely low level of CHL530 fluorescence emission that tracks with the CHL470 emission because Chlorophyll does absorb some green light (at 530 nm). However, in this case, with traces of Phytoplankton presence in the freshwater as indicated by the data count from the water body housing the fish-cages, there is a positive correlation values of 0.39 and 0.32 between the two key algae parameters (Chlorophyll-a (470) and Chlorophyll-a (530)), respectively, which is clearly demonstrated in both Table 2 and Fig. 6.

On the other hand, Turbidity maintains an extremely strong positive correlation of 0.9251 with CHL470 and 0.9741 with CHL530 as shown in both Table 2 and Fig. 6. This is also expected because the presence of biomass in freshwater tends to strongly affect the overall water colouration which can results in high level of Turbidity in the water body.

## Multivariate linear regression

Studies have shown that statistical methods like regression models are the best tools for studying any existing relationship between independent and dependent variables, especially with a diminutive data size^29,30^. The multivariate linear regression method is a technique widely applied to estimate any linear relationship that exists between one or more independent variables and a dependent variable. In this study, the multivariate linear regression was applied to model and establish the relationship between the multiple independent water quality parameters and the dependent parameter. A general multivariate linear regression equation represented by the model below was used:

$$Y={\beta }_{0}+{\beta }_{1}{X}_{1}+{\beta }_{2}{X}_{2}+{\beta }_{3}{X}_{3}+\cdots ++{\beta }_{i}{X}_{i}+\epsilon$$

where $Y$ denotes the dependent water quality parameter, $\beta$ represents the regression coefficients, ${X}_{1}, {X}_{2}, {X}_{3}, \ldots , {X}_{i}$ are the ${i}^{th}$ independent water quality parameters, and the error term is represented by $\epsilon$. For multiple observations, the multiple linear regression equation was rewritten as shown below:

$$\begin{aligned} & Y_{1} = \beta_{0} + \beta_{1} X_{11} + \beta_{2} X_{12} + \beta_{3} X_{13} + \cdots + + \beta_{i} X_{1i} + \epsilon \\ & Y_{2} = \beta_{0} + \beta_{1} X_{21} + \beta_{2} X_{22} + \beta_{3} X_{23} + \cdots + + \beta_{i} X_{2i} + \epsilon \\ & Y_{3} = \beta_{0} + \beta_{1} X_{31} + \beta_{2} X_{32} + \beta_{3} X_{33} + \cdots + + \beta_{i} X_{3i} + \epsilon \\ & \quad \quad \quad \quad \quad \quad \quad \quad \vdots \\ & Y_{j} = \beta_{0} + \beta_{1} X_{j1} + \beta_{2} X_{j2} + \beta_{3} X_{j3} + \cdots + + \beta_{i} X_{ij} + \epsilon_{i} \\ & \quad \quad \quad \quad \quad \quad \quad \quad \vdots \\ & Y_{n} = \beta_{0} + \beta_{1} X_{n1} + \beta_{2} X_{n2} + \beta_{3} X_{n3} + \cdots + + \beta_{i} X_{ni} + \epsilon_{n} \\ \end{aligned}$$

By applying Matrix form, the above multiple linear regression equations can be represented as shown below:

3 $$Y=X\beta +\epsilon$$

where

$$\begin{aligned} Y & = \left[ {\begin{array}{*{20}c} {Y_{1} } \\ {Y_{2} } \\ {Y_{3} } \\ \vdots \\ {Y_{n} } \\ \end{array} } \right],\;\;\;\;X = \left[ {\begin{array}{*{20}c} {\begin{array}{*{20}c} 1 & {X_{11} } & {X_{12} } \\ 1 & {X_{21} } & {X_{22} } \\ 1 & {X_{31} } & {X_{32} } \\ \end{array} \begin{array}{*{20}c} {X_{13} } & \cdots & {X_{1i} } \\ {X_{23} } & \cdots & {X_{2i} } \\ {X_{33} } & \cdots & {X_{3i} } \\ \end{array} } \\ {\begin{array}{*{20}c} \vdots & \vdots & \vdots \\ 1 & {X_{n1} } & {X_{n2} } \\ \end{array} \begin{array}{*{20}c} \vdots & \cdots & \vdots \\ {X_{n3} } & \cdots & {X_{ni} } \\ \end{array} } \\ \end{array} } \right] \\ \beta & = \left[ {\begin{array}{*{20}c} {\beta_{0} } \\ {\beta_{1} } \\ {\beta_{2} } \\ \vdots \\ {\beta_{i} } \\ \end{array} } \right],\;\;\;{\text{and}}\;\;\;Y = \left[ {\begin{array}{*{20}c} {\epsilon_{0} } \\ {\epsilon_{1} } \\ {\epsilon_{2} } \\ \vdots \\ {\epsilon_{n} } \\ \end{array} } \right] \\ \end{aligned}$$

The matrix $Y$ contains the information about the dependent water quality parameter and matrix $X$ contains the information about the independent water quality parameters. By applying the least square method^31^, the regression coefficients $\beta$ of equation $(3)$ can be obtained as shown below:

4 $$\beta ={X^\prime}Y{\left({X^\prime}X\right)}^{-1}$$

From the known regression coefficient $\beta$ in equation $(4)$, future water quality parameters can be predicted by applying the multivariate linear regression equation shown below in equation $(5)$:

5 $$\widehat{Y}=\beta X$$

Therefore, given a historical water quality parameters dataset, $\widehat{Y}$ becomes the prediction result of $Y$, where the difference between $Y$ and $\widehat{Y}$ is the prediction error which directly affects the overall prediction accuracy of the developed model. When the future independent water quality parameters matrix ${X}_{f}$ is collected, a prediction of the dependent water quality parameter ${Y}_{f}$ is obtained as shown in equation $(6)$ below:

6 $${Y}_{f}=\beta {X}_{f}$$

## Proposed hybrid forecasting model design

The EEMD method and deep learning LSTM NN were merged to form the Hybrid multivariate water quality parameters forecasting model. The basic implementation processes of EEMD method and LSTM deep learning NN technique are described in detail in Sections "EEMD method" and "Deep learning LSTM NNs", respectively.

### EEMD method

EEMD is a noise-assisted time-series dataset analysis method. In EEMD technique of time-series dataset analysis, Gaussian white noise is added to enable the separation of contrasting time-series scales, which in turn, leads to improved decomposition efficiency of the EMD method. The introduced white-noise comprises of components of disparate scale which would systematically fill the entire time–frequency space. The disparate scale components of the signal are spontaneously projected onto proper scales of reference initiated by the Gaussian white-noise as the systematically distributed white-noise is introduced to the signal. Since all the decomposed components of the introduced Gaussian white-noise consists of both the signal and the introduced white noise, all the individual trials usually end up with noisy results. However, the white-noise can be almost completely cancelled out with the aid of ensemble mean of whole trials because the white-noise in each of the trials are unique in different trials^27^. Therefore, the actual underlying components of the water quality time series data can be represented by the ensemble mean. In other words, EEMD method sums up the components and adopts the average as the true decomposition results. Finally, the result of decomposition solves the mode mixing drawbacks associated with conventional EMD method. It is a useful method for extracting underlying and crucial components from the water quality time series data.

For the CHL470, CHL530, and Turbidity time-series data, the EEMD method follows certain procedure which can be described as follows.

*Stage 1*: Initialize an ensemble number $M$ and the amplitude of the introduced Gaussian white-noise.

*Stage 2*: Perform the ${m}^{th}$ trial for introducing disparate white-noise ${W}_{m}\left(t\right)$ to $x\left(t\right)$ in order to generate the noise-augmented time series data ${x}_{m}\left(t\right)$, where

7 $${x}_{m}\left(t\right)=x\left(t\right)+{W}_{m}\left(t\right)$$

*Stage 3*: Determine all the local minima and maxima of ${x}_{m}\left(t\right)$ and use them to generate both lower and upper envelopes with the help of cubic spline interpolation functions.

*Stage 4*: Compute the mean ${m}_{1}\left(t\right)$ of both lower and upper envelopes.

*Stage 5*: Calculate the difference ${h}_{1}\left(t\right)$ that exists between the mean computed in stage 4 and the signal ${x}_{m}\left(t\right)$, using,

8 $${h}_{1}\left(t\right)={x}_{m}\left(t\right)-{m}_{1}\left(t\right)$$

*Stage 6*: If the properties of the intrinsic mode function (IMF) are satisfied by the ${h}_{1}\left(t\right)$, that is, from the signal ${x}_{m}\left(t\right)$, ${C}_{1}\left(t\right)={h}_{1}\left(t\right)$ becomes the first IMF component. Otherwise, replace ${x}_{m}\left(t\right)$ with ${h}_{1}\left(t\right)$ and return to Stage 3.

The two properties of IMF are described as follows: (i) the number of the zero crossing and extrema must either equal or differ at most by 1 over the entire data $x\left(t\right)$ and (ii) at any given point, the mean value ${h}_{1}\left(t\right)$ of the generated envelopes given by both local minimum and local maximum must be zero.

*Stage 7*: Separate the residue ${R}_{1}\left(t\right)$ from the rest of the dataset using,

9 $${R}_{1}\left(t\right)={x}_{m}\left(t\right)-{C}_{1}\left(t\right)$$

Let the residue ${R}_{1}\left(t\right)$ be a new signal and sift out the remaining IMFs by repeating Stage 3 through Stage 7 $n$ times until the stopping criterion is satisfied. The applied stopping criterion can be either of the following: (i) when the residue ${R}_{n}\left(t\right)$ is reduced to a monotonic function such that no more IMF can be extracted from it. (ii) when the residue ${R}_{n}\left(t\right)$ or IMF component ${C}_{1}\left(t\right)$ becomes smaller than the predetermined value. Then, after EEMD decomposition process, the original signal ${x}_{m}\left(t\right)$ can be mathematically expressed as the sum total of each of the IMFs ${C}_{1}\left(t\right)$ components and the residue ${R}_{1}\left(t\right)$. Hence,

10 $${x}_{m}\left(t\right)=\sum_{i=1}^{n}{C}_{i}\left(t\right)+{R}_{1}\left(t\right)$$

where $n$ and ${C}_{i}\left(t\right)$ denote total number of the IMFs ${C}_{1}\left(t\right)$ components and the ${i}^{th}$ IMF, respectively; and ${R}_{1}\left(t\right)$ represents the final residue.

Stage 8: By adding a different noise in each trial, repeatedly execute Stage 2 to Stage 7 until $m=M$ if $m<M$, through consecutive increment of the value of m by using $m=m+1$.

Stage 9: Determine the ${i}^{th}$ ensemble mean $\overline{{C }_{i}}$ of the M trials for individual IMF, by way of expression,

11 $$\overline{{C }_{i}}=\frac{1}{M}\sum_{m=1}^{M}{C}_{i},m i=1, 2, 3, \ldots , n$$

and the ensemble residue ${\overline{R} }_{n}$ can be expressed as

12 $${\overline{R} }_{n}=\frac{1}{M}\sum_{m=1}^{M}{R}_{n},m.$$

Therefore, the original Chlorophyll-a time series data is efficiently decomposed through EEMD method into $n$ ensemble IMFs and a single ensemble residue. In each frequency band, the contained IMF components are individually different and can change with the variation of the Chlorophyll-a time series dataset $x\left(t\right)$. Additionally, the ensemble residue denotes the general trend of the Chlorophyll-a dataset $x\left(t\right)$.

### Deep learning LSTM NNs

**Figure 7:**
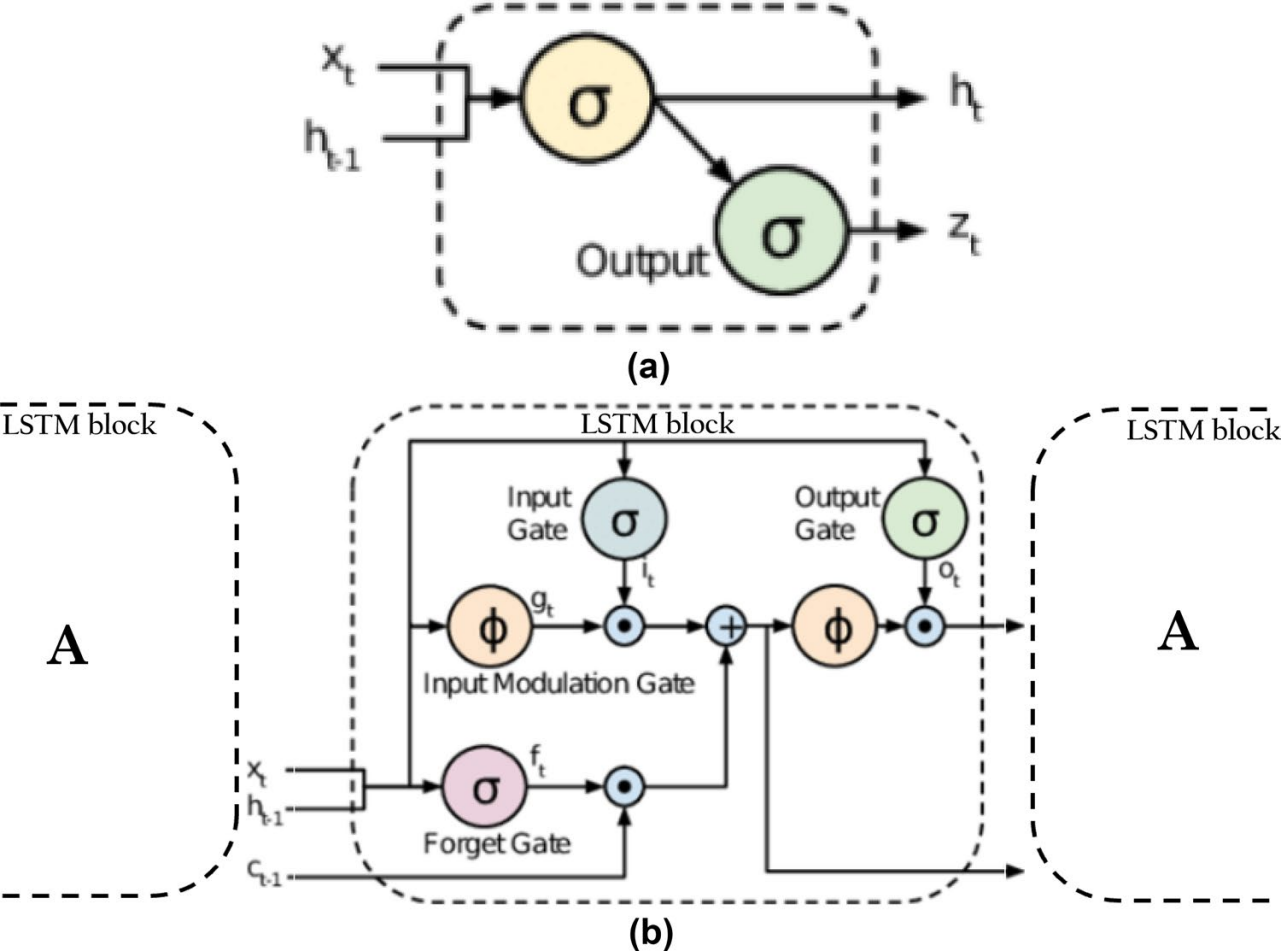
(**a–b**) Typical schematic diagram of (**a**) Traditional RNN node, and (**b**) Chained (deep learning) LSTM blocks.

Deep Learning LSTM NN is a special type of Recurrent NN (RNN) with significant improvement and the ability to learn long-term dependencies which gives it an advantage over other ANNs such as BPNN, RBFNN, etc. RNN is a deep learning model specifically designed to handle the analysis and processing time-series datasets. Figure 7a and b illustrate typical schematic diagrams of traditional RNN node and deep learning LSTM NN, respectively, with the previous hidden state represented by ${h}_{t-1}$, activation tanh function, current input sample by ${X}_{t}$, current output by ${h}_{t}$, and the current hidden state by ${h}_{t}$. As depicted in Fig. 7a, all RNNs generally have the form of a chain repeating modules of NNs. These repeating modules generally have a very basic structure in standard RNNs like a single tanh layer only. However, deep learning LSTM which stores information with the aid of purpose-built memory cells maintains similar chain-like structure, but with a different structured repeating module (see Fig. 7b). As illustrated in Fig. 7b, there are four distinct interacting layers in deep learning LSTM architecture^32^. Equations below illustrates the calculation processes involved in deep learning LSTM NN architecture.

- Forget gate equation:
  13 $${F}_{t}=\sigma \left({W}_{f}\times \left[{h}_{t-1},{X}_{t}\right]+{b}_{f}\right)$$
  where ${F}_{t}$ is a vector with values from 0 to 1, with $\sigma$, ${W}_{f}$, and ${b}_{f}$ represent the logistic sigmoid function, weight matrices and bias of the forget gate, respectively. The sigmoid layer determines if the new information is necessary to be used for update or unnecessary and ignored. Then, tanh function adds weight to each value that passed and decides their level of importance ranging from − 1 to 1. Similar operations are repeated in input and output gates shown in (14) through (17).
- Input gate equations:
  14 $${I}_{t}=\sigma \left({W}_{i}\times \left[{h}_{t-1},{X}_{t}\right]+{b}_{i}\right)$$
  15 $${\widehat{I}}_{t}=\mathrm{tanh}\left({W}_{i}\times \left[{h}_{t-1},{X}_{t}\right]+{b}_{i}\right)$$
- Output gate equations:
  16 $${O}_{t}=\sigma \left({W}_{o}\times \left[{h}_{t-1},{X}_{t}\right]+{b}_{o}\right)$$
  17 $${h}_{t}={O}_{t}\times \mathrm{tanh}\left({C}_{t}\right)$$
- Cell state equation:
  18 $${C}_{t}=\left\{\left({F}_{t}\times {C}_{t-1}\right)+\left({I}_{t}\times {\widehat{I}}_{t}\right)\right\}$$
  where ${W}_{i}$ and ${W}_{o}$ denote the weight matrixes, ${b}_{i}$ and ${b}_{o}$ represent the network’s bias vectors, of the input and output gates. Tanh represents the hyperbolic tangent function.

### Hybrid water quality parameters forecasting model

**Figure 8:**
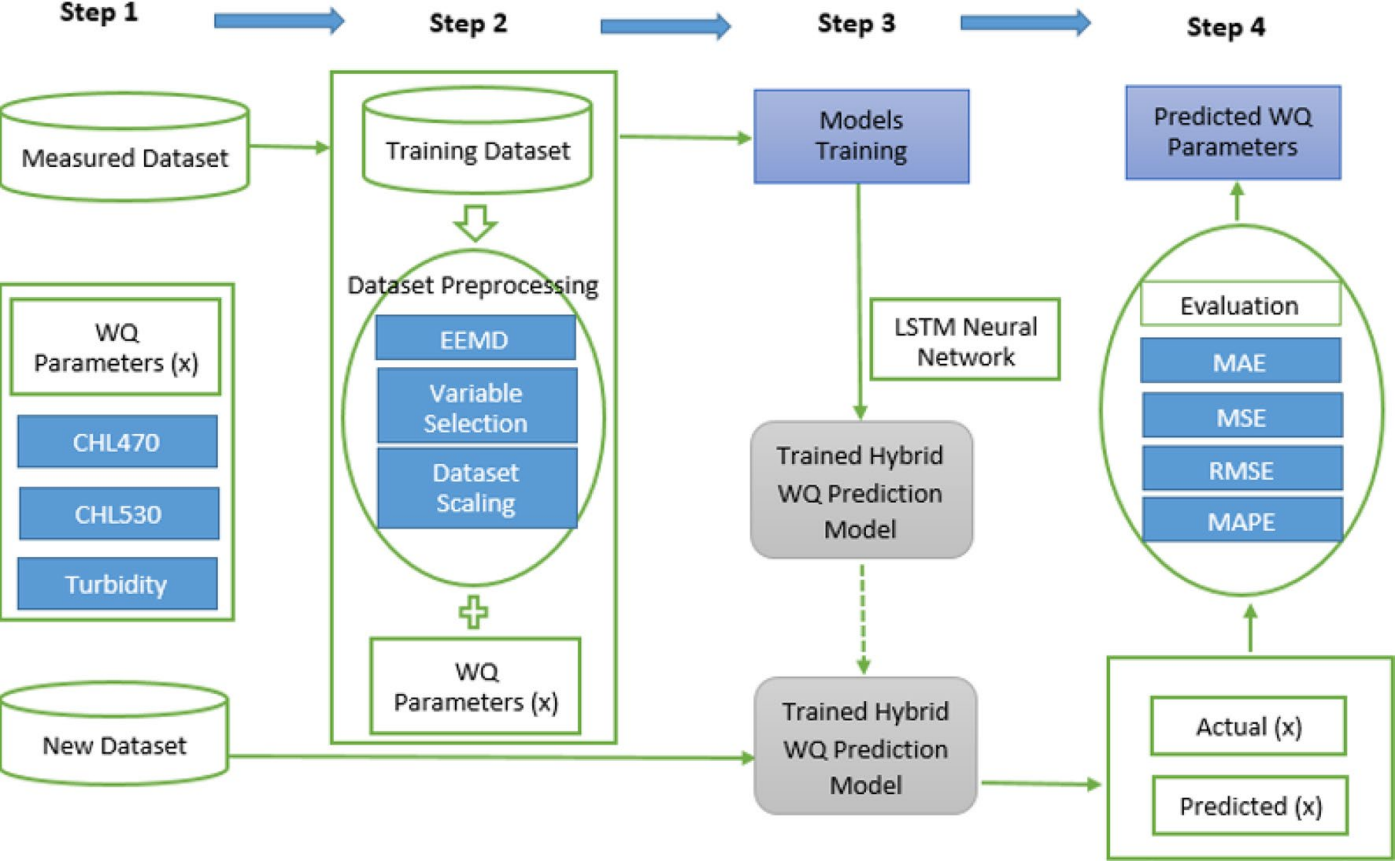
Proposed hybrid water quality parameters forecasting model.

The proposed hybrid EEMD-LSTM deep learning NN based water quality parameters forecasting model is shown in Fig. 8. With the proposed novel water quality prediction model, measured real water quality parameters concentration data set is first decomposed through EEMD method into several components to improve the prediction accuracy of the proposed model. The detailed procedures demonstrated in Fig. 8 shows the four crucial stages that lead to the development of the new hybrid EEMD-LSTM based water quality parameters prediction Model. In the first stage (stage 1), water quality parameters time series data $x\left(t\right)$ is pre-processed, followed by the decomposition of $x\left(t\right)$ into several IMFs and a residual item ${R}_{N}(t)$ in stage 2 by the applied EEMD algorithm in the input layer of the deep learning LSTM NN. The data set decomposition is performed through an iterative sifting process which is expressed as

19 $$x\left(t\right)=\sum_{i=1}^{N}{IMF}_{i}\left(t\right)+{R}_{N}\left(t\right).$$

Then, each IMF and residual item is normalized and used for forecasting at the hidden layer of the deep learning LSTM NN as demonstrated in Fig. 8. Finally, reverse normalization of individual forecast results of the deep learning LSTM NN is carried out prior to efficiently combine all of them together through summation operation with the aid of summation function to get the final predicted values in the output layer of the NN as shown in stage 4 of Fig. 8. In stage 3 of the proposed hybrid forecasting model, there are multiple hidden layers in the LSTM (LSTM_1,1_, LSTM_1,2_, …, LSTM_m,1_, up to LSTM_m,n_). Individual hidden layer of the stacked LSTM is equipped with numerous memory cells which earns the proposed forecasting model *deep learning* NN technique.

## Performance evaluation metrics

For the evaluation of the proposed hybrid EEMD-LSTM deep learning water quality prediction model, four performance evaluation metrics were introduced to evaluate its prediction accuracy. These metrics include MAE, MSE, RMSE, and MAPE. The mathematical formulae are expressed as follows:

20 $$\mathrm{MAE}=\frac{1}{n}\sum_{i=1}^{n}\left|{M}_{i}-{F}_{i}\right|$$

21 $$\mathrm{MSE}=\frac{1}{n}\sum_{i=1}^{n}{\left({M}_{i}-{F}_{i}\right)}^{2}$$

22 $$\mathrm{RMSE}=\sqrt{\frac{1}{n}\sum_{i=1}^{n}{\left({M}_{i}-{F}_{i}\right)}^{2}}$$

23 $$\mathrm{MAPE}=\frac{1}{n}\sum_{i=1}^{n}\left|\frac{{M}_{i}-{F}_{i}}{{M}_{i}}\right|.$$

In (20), (21), (22), and (23) above, $n$ denotes the number of data points in the dataset, ${M}_{i}$ and ${F}_{i}$ represent the measured real values and the predicted values, respectively. The closer the values of these four performance evaluation metrics tend towards 0, the higher the overall prediction and fitting accuracy of the proposed model.

## Results and discussions

**Figure 9:**
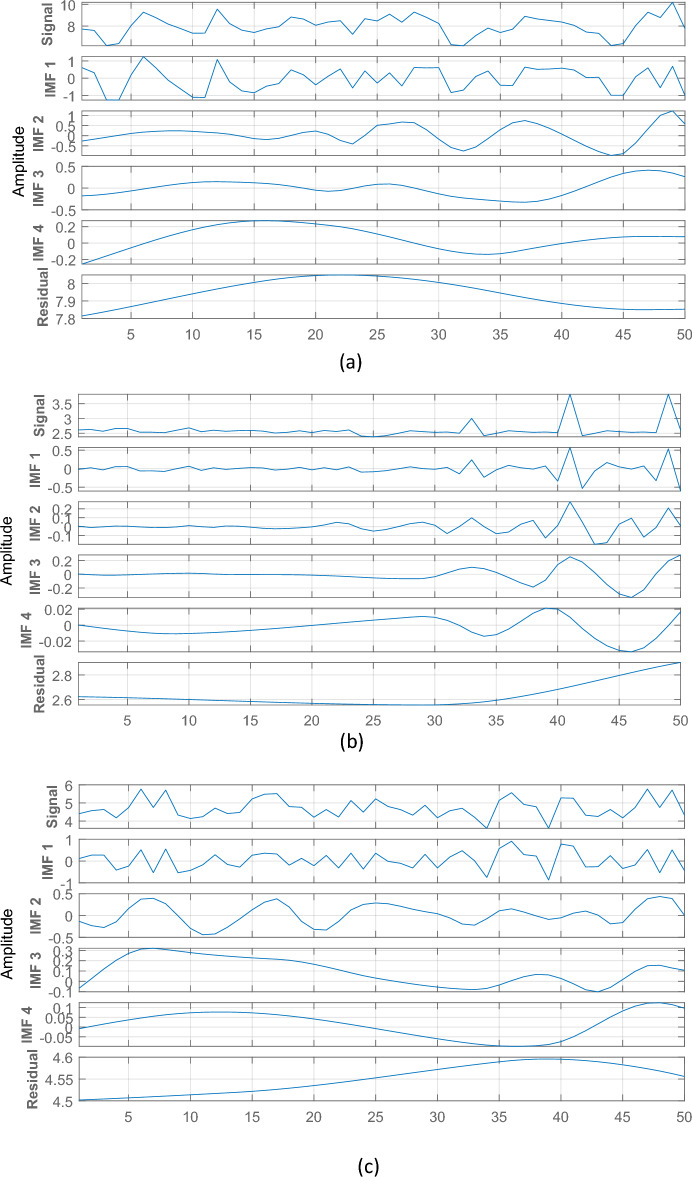
Independent water quality parameters dataset decomposition results for: (**a**) Chlorophyll-a (470), (**b**) Turbidity, and (**c**) Chlorophyll-a (530).

An hourly centred moving average values is applied in this study to the real water quality parameters time-series dataset from Loch Duart Salmon offshore aquaculture farms. Additionally, decomposing the TriLux multi-parameter sensor measured Chlorophyll-a (470), Turbidity, and Chlorophyll-a (530) contents time-series data with the EEMD technique is an integral part of the developed novel hybrid forecasting model. The EEMD method is a reliable and efficient technique for non-stationary, non-linear time-series signal decomposition. The steps involved in EEMD technique of time-series data signal decomposition processes as described in Section "EEMD method" decomposes the real measured Chlorophyll-a (470), Turbidity, and Chlorophyll-a (530) concentration sensor time-series data signals into four (4) relatively stable IMFs (IMF 1–4) and one residual item as shown in Fig. 9. All the obtained different stable IMFs and the corresponding residue from the original Chlorophyll-a (470), Turbidity, and Chlorophyll-a (530) data signal decomposition with EEMD method is shown in Fig. 9a–c. For an improved forecasting performance, the amplitude of the added White-Gaussian noise in the EEMD process was set to 0.2^33^. During the data signals decomposition process, summation of the low-frequency IMFs was used to extract the EEMD trend. Finally, the EEMD technique extracts strongly correlated set of sub-band signals which are used in decomposition process of the novel hybrid forecasting model.

**Figure 10:**
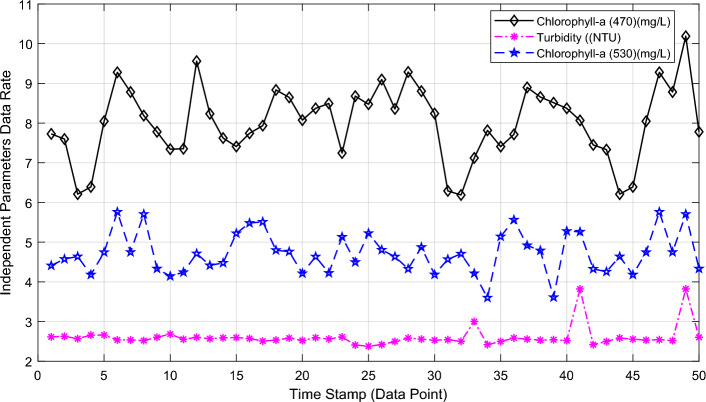
The three independent water quality parameters—Chlorophyll-a (470), Turbidity, and Chlorophyll-a (530).

The pre-processed real measured dataset is divided into two sets: seventy-five percent (75%) as a learning data sample (training dataset) and twenty-five percent (25%) for testing of the proposed novel hybrid forecasting model. Figure 10 shows the actual measured independent water quality parameters containing Chlorophyll-a (470), Turbidity, and Chlorophyll-a (530) after pre-processing.

**Figure 11:**
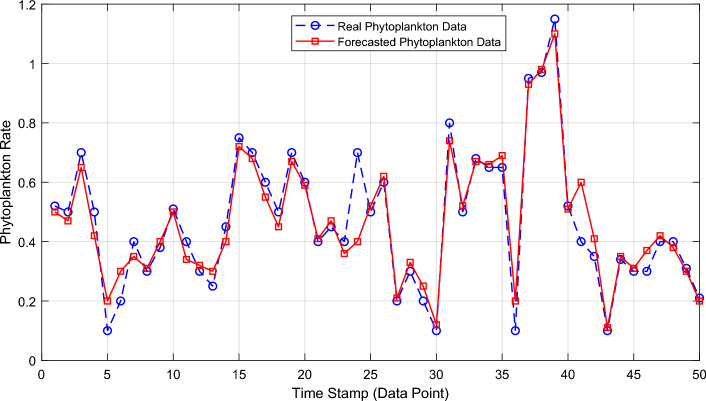
Comparison of the real and forecasted Phytoplankton data (dependent water quality parameter).

The forecast results were compared with the real monitored water quality parameters data from the Salmon offshore aquaculture farms. Figure 11 presents the achieved result showing the outcome of the novel hybrid forecasting model. The comparison of the forecasted Phytoplankton data with the real Phytoplankton data obtained from laboratory green biomass data count from Loch Duart Salmon offshore aquaculture farms as demonstrated in Fig. 11 clearly show that the novel hybrid forecasting model provided good results for the forecast horizon that covers the existing 50 Phytoplankton data points. With the actual measured independent water quality parameters concentration dataset containing Chlorophyll-a (470), Turbidity, and Chlorophyll-a (530), the matching trends between the real and forecasted Phytoplankton data points as shown in Fig. 11 further indicates that the proposed model can successfully forecast, with a high-level of accuracy, the presence of algal bacterial in aquaculture ecosystem.

**Figure 12:**
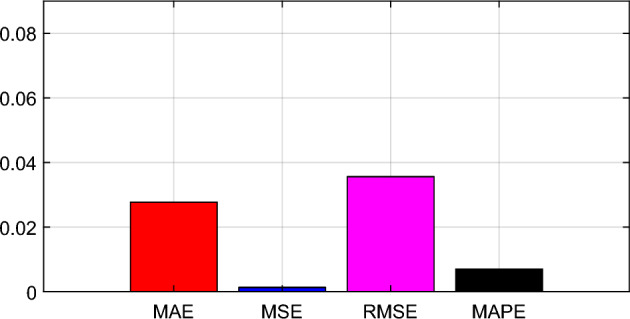
Bar Chart representation of the forecast error statistics for the proposed novel hybrid model.

The improved forecasting accuracy of the proposed novel hybrid forecasting model is due to the applied EEMD technique which allows the forecasting model to reflect the temporal characteristics of the measured time-series Chlorophyll-a (470), Turbidity, and Chlorophyll-a (530) concentration dataset. This is achieved with the aid of the multi-feature selection process used by the EEMD technique which enables the selection of a set of stable IMFs which strongly correlate with the actual measured Chlorophyll-a (470), Turbidity, and Chlorophyll-a (530) data and integrate them into inputs for the deep learning LSTM NN. The forecast error statistics of the proposed novel hybrid model were obtained from (20), (21), (22), and (23) for MAE, MSE, RMSE, and MAPE, respectively, as shown in Table 3 and Fig. 12. These marginal errors have further demonstrated the efficiency and reliability of the proposed novel hybrid model. However, the overall forecasting accuracy of the proposed novel hybrid model could be further improved with increased data availability because the complex chain structure of the deep learning LSTM NN tends to perform even better with Big data.

**Table 3:** Forecast error statistics for the proposed novel hybrid model.

Error statistics	6 hour prediction
MAE	0.0375
MSE	0.0024
RMSE	0.0489
MAPE	0.0072

In Table 4, the performance of the proposed hybrid EEMD–MLR–LSTM NN model is compared with other closely related hybrid water quality forecasting models based on SAE-LSTM NN, SAE-BPNN, single LSTM and BPNN^15^. The tabulated error statistics indicate that our proposed novel hybrid EEMD–MLR–LSTM NN model outperformed the other closely related hybrid models as shown in Table 4 in terms of the error margin of the predicted data. This performance gain over the other related hybrid prediction models is because our proposed hybrid EEMD–MLR–LSTM NN model applied the high potential EEMD method which allows for effective decomposition of the original data signal into its constituent multiple intrinsic sub-sequences. Consequently, our proposed hybrid, multi-scale EEMD–MLR–LSTM NN model can get more features through the decomposition process for the predicted data signals, which further leads to improved prediction accuracy of the model as opposed to the other closely related hybrid models. Among the similar water quality prediction models proposed in^15^, the hybrid SAE-LSTM model demonstrated the least error in terms of prediction accuracy. However, the tabulated error statistics in Table 4 indicate that our proposed novel hybrid EEMD–MLR–LSTM NN model outperformed the hybrid SAE-LSTM model due to the potentials of the applied EEMD technique.

**Table 4:** Performance comparison with closely related water quality forecasting models.

Error Statistics	LSTM NN	BPNN	SAE-LSTM NN	SAE-BPNN	EEMD–MLR–LSTM NN
Run Time (s)	23.2	3.6	29.6	9.1	3.7
MAE	0.1590	0.4530	0.1260	0.4060	0.0375
MSE	0.0398	0.3013	0.0242	0.2428	0.0024
RMSE	0.1995	0.5489	0.1556	0.4927	0.0489
MAPE	0.0160	0.0450	0.0130	0.0419	0.0072

## Conclusion

This study presents the development of a novel hybrid water quality forecasting model based on monitored TriLux multi-parameter sensor water quality parameters through the application of specialised EEMD method, MLR, and deep learning LSTM NN. The actual experimental real water quality data from Loch Duart Salmon aquaculture farms show that the proposed model provides useful future water condition forecast outcome with high accuracy. The forecast result in Fig. 11 has indicated and buttressed the importance of applying the proposed novel hybrid EEMD–MLR–LSTM NN model to aquaculture water quality management. It also shows that early forecasting of harmful green biomass (Algal blooms) with the aid of the actual TriLux multi-parameters sensor-monitored Chlorophyll-a (470), Turbidity, and Chlorophyll-a (530) contents time-series data in freshwater ecosystem can provide useful information for the effective operation and management of aquaculture industry. For future work, more water quality parameters measuring sites will also be considered to expand the proposed model.
